# Supplementary material for: Ultra-processed food consumption and the risk of overweight and obesity in adolescents: A systematic review and meta-analysis
Source: PLoS One. 2026 Apr 15;21(4):e0344873. doi: 10.1371/journal.pone.0344873 (PMC13082653; doi:10.1371/journal.pone.0344873)
Supplement: S1 File — (DOCX) [file pone.0344873.s001.docx]

**Supplementary 3. Ultra-Processed Food Consumption and the Risk of Overweight and Obesity in Adolescents: A Systematic Review and Meta-Analysis**

| ***Type of database*** | ***Number of articles identified*** | | ***Search Terms For Ultra-Processed Food Consumption and the Risk of Overweight and Obesity in Adolescents: A Systematic Review and Meta-Analysis*** | ***Accessed Date and time*** |
| --- | --- | --- | --- | --- |
|  |  | |  |  |
| ***Pub med*** | ***1,874*** | ***1*** | ***("ultra processed"[Title/Abstract] OR "ultraprocessed"[Title/Abstract] OR "ultra-processed"[Title/Abstract] OR*** ***" Processed  Food "[Title/Abstract] OR "industrialized"[Title/Abstract] OR "fast-food"[Title/Abstract] OR "fast food"[Title/Abstract] OR "fastfood"[Title/Abstract] OR "junk food"[Title/Abstract] OR "carbonated beverage"[Title/Abstract] OR "soft drink"[Title/Abstract] OR "sweetened beverage"[Title/Abstract] OR "sausage"[Title/Abstract])*** | ***July 25, 2025***  ***03:42:10*** |
|  |  | ***2*** | ***("Adolescent"[MeSH Terms] OR "adolescents"[Title/Abstract] OR "teenagers"[Title/Abstract] OR "youth"[Title/Abstract])*** ***OR ("Teen"[Title/Abstract] OR "Teenager"[Title/Abstract] OR "Young People"[Title/Abstract] OR "Young Person"[Title/Abstract])*** |  |
|  |  | ***3*** | ***("Obesity"[MeSH Terms] OR "Overweight"[MeSH Terms] OR "Body Mass Index"[MeSH Terms] OR "obesity"[Title/Abstract] OR "overweight"[Title/Abstract] OR "BMI"[Title/Abstract])*** |  |
|  |  | ***4*** | ***#1 AND #2 AND #3*** |  |
| ***Hinari*** | ***114*** | | *(TitleCombined:(\("ultra processed" OR "ultraprocessed" OR "ultra-processed" OR "processed food" OR "industrialized" OR "fast-food" OR "fast food" OR "fastfood" OR "junk food" OR "carbonated beverage" OR "soft drink" OR "sweetened beverage" OR "sausage")) AND (TitleCombined:("adolescents" OR "teen" OR "teenager" OR "teenagers" OR "youth" OR "young people" OR "young person")) AND (TitleCombined:("obesity" OR "overweight" OR "BMI" OR "Body Mass Index"))* | ***July 25, 2025***  ***5:49*** |
| ***Science direct*** | ***144*** | | *("ultra processed" ) AND (adolescent OR adolescents OR teenager ) AND (obesity OR overweight) NOT Adult* | ***July 25, 2025***  ***5:49*** |
| ***google scholar*** | ***15*** | |  | ***July 30, 2025*** |
| ***Total*** | ***2100*** | |  |  |
