# Supplementary material for: Ultra-processed food consumption and the risk of overweight and obesity in adolescents: A systematic review and meta-analysis
Source: PLoS One. 2026 Apr 15;21(4):e0344873. doi: 10.1371/journal.pone.0344873 (PMC13082653; doi:10.1371/journal.pone.0344873)
Supplement: S3 File — (DOCX) [file pone.0344873.s003.docx]

Supplementary 3. Methodological quality assessment of included studies using Newcastle-Ottawa Scale (NOS) for Vitamin D defiecency in SSA, 2025

| **No.** | **Author/year** | **Representativeness** | **Sample size** | **Non-respondents** | **Ascertainment of the exposure** | **comparability** | **Assessment of outcome** | **Statistical test** | **Quality score** |
| --- | --- | --- | --- | --- | --- | --- | --- | --- | --- |
| 1 | Alam et al. | 1 | 0 | 0 | 1 | 1 | 2 | 1 | 6 |
| 2 | B. Saleh and E. Ma΄ala | 1 | 1 | 1 | 1 | 1 | 2 | 1 | 8 |
| 3 | Banik et al. | 1 | 1 | 1 | 1 | 1 | 2 | 1 | 8 |
| 4 | Borges et al. | 1 | 1 | 1 | 1 | 1 | 2 | 1 | 8 |
| 5 | Chernet Elias et al. | 1 | 1 | 1 | 1 | 1 | 2 | 1 | 8 |
| 6 | Denova-Gutiérrez E. et al. | 1 | 1 | 0 | 1 | 1 | 2 | 1 | 7 |
| 7 | Francis DK et al. | 1 | 1 | 1 | 1 | 1 | 2 | 1 | 8 |
| 18 | Goyal et al. | 1 | 1 | 1 | 1 | 1 | 2 | 1 | 8 |
| 9 | Kerkadi et al | 1 | 1 | 1 | 1 | 1 | 2 | 1 | 8 |
| 10 | Khan et al. | 1 | 1 | 1 | 1 | 1 | 2 | 1 | 8 |
| 11 | M. Anitha Rani | 1 | 1 | 1 | 1 | 1 | 2 | 1 | 8 |
| 12 | Makri et al. | 1 | 1 | 1 | 1 | 1 | 2 | 1 | 8 |
| 13 | Neri, D. et al. | 1 | 1 | 1 | 1 | 1 | 2 | 1 | 8 |
| 14 | Nguyen et al. | 1 | 1 | 1 | 1 | 1 | 2 | 1 | 8 |
| 15 | Putra and Santoso | 1 | 0 | 1 | 1 | 1 | 2 | 1 | 7 |
| 16 | Ra, J. S. & Huyen, D. T. T | 1 | 1 | 1 | 1 | 1 | 2 | 1 | 8 |
| 17 | Rifqi et al | 1 | 0 | 0 | 1 | 1 | 2 | 1 | 6 |
| 18 | Souza SF. et al. | 1 | 1 | 1 | 1 | 1 | 2 | 1 | 8 |
| 19 | Stanislas and Santoso | 1 | 0 | 1 | 1 | 1 | 2 | 1 | 7 |
| 20 | Vanitha et al. | 1 | 0 | 1 | 1 | 1 | 2 | 1 | 7 |
| 21 | Wuenstel, J. W. et al. | 1 | 1 | 1 | 1 | 1 | 2 | 1 | 8 |
| 22 | Yolanda Patrichia et al. | 1 | 0 | 1 | 1 | 1 | 2 | 1 | 7 |
| 23 | Zhihao Huang et al. | 1 | 1 | 1 | 1 | 1 | 2 | 1 | 8 |
