# Supplementary material for: Ultra-processed food consumption and the risk of overweight and obesity in adolescents: A systematic review and meta-analysis
Source: PLoS One. 2026 Apr 15;21(4):e0344873. doi: 10.1371/journal.pone.0344873 (PMC13082653; doi:10.1371/journal.pone.0344873)
Supplement: Attachment — Submitted filename: Resposnse_to_reviewer_auresp_2.docx [file pone.0344873.s007.docx]

**18 Feb, 2026**

**Dear Editors of the Plos One,**

We are grateful for the time and expertise devoted to reviewing manuscript PONE-D-25-46416 titled “Ultra-Processed Food Consumption and the Risk of Overweight and Obesity in Adolescents: A Systematic Review and Meta-Analysis”. We sincerely appreciate the reviewers’ constructive feedback, which has helped us improve the scientific clarity and overall quality of the paper.

In response, we have carefully revised the manuscript and provided a detailed, point-by-point reply outlining how each comment has been addressed. Revisions within the manuscript are clearly marked for easy review. We believe these changes strengthen the analysis, interpretation, and presentation of our findings.

Thank you for the opportunity to revise our work.

**Warm regards,**

**Mekuriaw Nibret Aweke**

**Corresponding Author**

1. **Editors comment**
1. Please address the Reviewer #2 suggestions

Authers response:

Dear Editor,

We are very pleased to have recived the quick feedback from the editor and reviewers after revision. Accordingly we have corrected and revised the manuscript based on the suggesions of reviewer #2. Thank you very much for the opportunity .

2. Please formally justify the inclusion of the new Author in the co-authors list

Authers response:

We respectfully request approval to add **Anas Ali Alhur** to the list of authors for this manuscript. The Authors had substantial contributions to analysis, interpretation of data critically revising the manuscript for important intellectual content; and final approval of the version to be published.

He also provided **critical intellectual revisions** to the manuscript that enhanced its scientific rigor and clarity. **Anas Ali Alhur** has reviewed and approved the final version of the manuscript and agrees to be accountable for all aspects of the work, ensuring the accuracy and integrity of the research. This authorship update does not affect the study’s results, conclusions, or disclosures, and no conflicts of interest arise from this change.

2. **Revewer #1 comments**

The authors have satisfactorily addressed all the questions and comments raised in my previous review, and I believe the manuscript is now suitable for publication. However, I would like to point out that Figure 8 still lacks a legend and the axes are not defined, which makes the figure difficult to interpret. I recommend addressing this minor issue before final acceptance.

**Response to Reviewer:**

Dear reviewer thank you very much for your acceptance that we have addressed the questions and comments. In addition we have revised figure to by including legend and defined the axes which will now easy to interpret.

We appreciate your invaluable feedback to enhance the quality of manuscript.

3. **Reviewer 2 comments and responses**

The manuscript appears to have improved following the reviewers’ suggestions.

Author response

Dear Reviewer, we are very glad to hear this positive feedback about the improvement of the manuscript after reviewers suggesions.

**Comment 1.** In my opinion, the decision to include all classifications available in the literature to categorise UPFs necessitates the provision of additional methodological detail. First, I recommend adding a new table (similar to Table 2) that includes a column describing the method used to classify UPFs in each study included in the review. Furthermore, a similar level of detail should be provided for the outcomes, specifying the methods used to assess overweight and obesity. The proposed table could include the following columns: S. No., Author, Exposure Assessment, Outcome Assessment, Results, and Conclusion.

Author response

Dear Reviewer, thank you for this valuable comment. We agree that providing additional methodological detail strengthens the clarity and transparency of the review. Accordingly, we revised the manuscript by adding a new table (Table 3), structured similarly to Table 2, which explicitly details the methods used to classify ultra-processed foods in each included study. This table now includes information on the exposure assessment (including UPF classification approaches), outcome assessment methods for overweight and obesity, as well as the corresponding results and conclusions of each study. These revisions enhance comparability across studies and improve the methodological rigor of the review.

**Comment 2** **Finally, the statement that the use of the Nova system represents a strength of this review (lines 352–355) should be supported strictly by the results obtained. Such a claim should be made cautiously and only if clearly justified by the findings**.

Author response

Dear Reviewer, thank you for this important comment. We agree that claims regarding methodological strengths should be made cautiously and be directly supported by the findings. Accordingly, we have removed the statement asserting the use of the NOVA classification as a strength of the review (formerly lines 352–355) to avoid overstating its contribution beyond what is clearly justified by the results.

**Comment 3** In conclusion, in addition to the suggestions mentioned above to improve the scientific quality of the manuscript, I strongly recommend that the authors seek professional English language editing prior to resubmission. The manuscript contains frequent grammatical errors, awkward sentence constructions, and typographical mistakes, which significantly affect its overall clarity..
Author response

Dear reviewer, we thank the reviewer for this valuable suggestion. We have revised spelling errors typographical and other mistakes and we hope this revision will improve the updated version of the manusrcipt.
